# Improvement of Soluble Expression, Stability, and Activity of Acetaldehyde Lyase by Elastin-like Polypeptides Fusion for Acetoin Production from Acetaldehyde

**DOI:** 10.3390/biom15091216

**Published:** 2025-08-22

**Authors:** Hui Lin, Jiming Zhang, Jie Hu, Lu Ma, Kaili Lai, Chaosong Zheng, Qiuhua Yang, Liaoyuan Zhang

**Affiliations:** 1 Institute of Edible Fungi, Fujian Academy of Agricultural Sciences, Fuzhou 350002, China; linhui-syjs@faas.cn (H.L.);; 2 College of Life Sciences, Fujian Agriculture and Forestry University, Fuzhou 350002, China; 3 College of Food Science, Fujian Agriculture and Forestry University, Fuzhou 350002, China; 4 Fisheries Research Institute of Fujian, Xiamen 361013, China

**Keywords:** elastin-like polypeptides, acetaldehyde lyase, non-chromatographic purification, stability and substrate tolerance, acetoin production

## Abstract

To achieve the large-scale, low-cost preparation of acetaldehyde lyase (ALS), elastin-like polypeptides (ELPs) as non-chromatographic purification tags were employed to develop an ELP-ALS fusion protein in *Escherichia coli*. Induction expression results demonstrated that the ELPs tag efficiently improved the soluble expression of the ALS enzyme. Through two rounds of inverse transition cycling (ITC), highly pure ELP-ALS was obtained with an enzyme recovery rate of 85.77%, outperforming Ni^2+^-affinity chromatography (66.80%). The comparative analysis of enzymatic properties revealed that ELP fusion markedly improved the stability and substrate tolerance of the ALS enzyme. Kinetic parameter analysis under identical conditions showed that ELP-ALS possessed a *V*_max_ of 15.25 U/mg and a *k*_cat_/*K*_m_ of 73.05 s^−1^·M^−1^, representing 1.86-fold and 2.97-fold improvements over His-ALS, respectively. Fed-batch reaction using ELP-ALS and acetaldehyde as biocatalyst and substrate, respectively, yielded 95.92 g/L acetoin with 49.32% increase compared to His-ALS (64.24 g/L). These results demonstrated the application potential of ELP-ALS as a promising biocatalyst for acetoin production from acetaldehyde due to its lower preparation cost, higher biocatalytic efficiency, better stability, and substrate tolerance.

## 1. Introduction

The global environmental challenges, including global warming, environmental pollution, depleting fossil stocks, and the degradation of natural habitats and biodiversity, are daunting. Tradition artificial chemical synthesis methods significantly contribute to these issues, driving increasing attention toward green biosynthesis technologies [1]. Biocatalysis, a key component of “green chemistry”, enables environmentally friendly industrial processes that maximize resource utilization and minimize waste generation [2], aligning with the Sustainable Development Goals (SDGs) of the EU and global initiatives [3]. Over the past two decades, biocatalysis has matured into a widely adopted technology, with industrial-scale applications expanding exponentially. Compared to conventional chemical processes, biocatalysis offers a powerful and sustainable approach for synthesizing diverse chemicals. It is now extensively applied in industries such as food, pharmaceuticals, biofuels, and detergents through whole-cell catalysis and cell-free systems [4,5]. However, challenges persist in catalytic efficiency and enzyme stability.

Acetoin (3-hydroxybutanone), a naturally occurring compound with a yogurt-like aroma and creamy flavor, is widely used in agriculture, food, cosmetics, and chemical industries [6]. For example, in the food industry, it is commonly added as a natural flavor enhancer of cream, yogurt, coffee, and cheese. As for yeast-fermented products, acetoin exists in almost all alcoholic beverages and is indispensable in contributing to their flavors [7]. In agricultural applications, this compound functions as a potent bioactive agent to enhance plant growth and development through hormonal signaling pathways [8,9]. As a platform chemical, it can be further refined via the production of butanediols or butanols and used for instance as precursor for pharmaceutical *R*-enantiomer, Tetramethylpyrazine, or fuel production [10,11]. Recognized by the U.S. Department of Energy as one of the 30 priority platform compounds, its global market value reached USD 177.01 million in 2022, projected to grow to USD 236.41 million by 2028 [12]. Most commercial acetoin is chemically synthesized, a process associated with environmental harm, low yield, and safety concerns, particularly in food and cosmetics sectors [13]. Depleting fossil resources further challenge traditional methods. Biotechnology driven approaches, including microbial fermentation, whole-cell biocatalysis, and cell-free catalysis, have emerged as sustainable alternatives [14]. Acetoin is naturally produced by various microorganisms such as *Saccharomyces cerevisiae* [15], *Candida glabrata* [16], *Escherichia coli* [17], *Serratia marcescens* [18], *Klebsiella pneumoniae* [19], *Corynebacterium glutamicum* [20], *Bacillus amyloliquefaciens* [21], and *Bacillus subtilis* [22], among others. Microbial fermentation achieves high acetoin titers by development of novel fermentation strategies and engineered strains, but challenges including byproduct formation and purification complexity need to be overcome [23]. Enzyme catalysis can mitigate these downstream challenges and offers several advantages, such as controllable reaction conditions, rapid reaction rate, high product yield, and easier process optimization [24]. Whole-cell catalysis stabilizes enzymes but faces mass transfer limitations due to cell membranes, which hinder substrate/product diffusion and reaction rates [25]. Cell-free systems circumvent these issues, enabling rapid kinetics and high purity, making them a research focus.

Today, using a multi-enzyme cell-free transformation system, inexpensive substrates like ethanol, pyruvic acid, and xylose have been successfully converted into acetoin [26,27,28]. But the yield of acetoin remains relatively low, and traditional enzymes often struggle to meet efficiency demands. The development of a new C-C bond forming enzyme formolase (FLS) from *Pseudomonas fluorescens* has emerged as a standout due to its exceptional catalytic activity for carbon ligation of acetaldehyde [29,30]. In our previous work, we conducted a proof-of-concept biosynthesis of acetoin and 2,3-butanediol from ethanol using a multi-enzyme synthetic biosystem. Unfortunately, the relatively slow catalytic efficiency of FLS was identified as the rate-limiting step [31]. Consequently, our subsequent research focused on systematically modifying FLS. The rational engineering of FLS’s substrate-binding pocket and tunnel yielded the mutant FLS: I28V/L482E with improved efficiency [32]. Typically, His-tags have been introduced into recombinant FLS: I28V/L482E to facilitate the subsequent purification of the enzyme. However, poor soluble expression in *Escherichia coli* and inefficient purification by Ni-NTA hindered its industrial application prospects. Acidic fusion tags have attracted interest as an effective method to address this issue.

Elastin-like polypeptides (ELPs), artificial thermally responsive biopolymers, are composed of repetitive sequences Valine-Proline-Glycine-Xaa-Glycine (GenBank: QWF36692.1), where Xaa can be any amino acid except for proline [33]. ELPs exhibit rapid and thermodynamically reversible phase transition behavior at a specific temperature known as the inverse transition temperature (T*_t_*). This transition can also be triggered isothermally by decreasing T*_t_* below the solution temperature through salt addition. The inverse transition cycling (ITC) technique, which is simple, rapid, and scalable, exploits acidic fusion tag ELP’s thermally responsive properties for the purification of recombinant proteins [34,35]. To address the solubility and preparation difficulties associated with FLS: I28V/L482E (designated as ALS in this article), ELP, as a purification tag, was fused with ALS, yielding the ELP-ALS protein, which can be easily purified through ITC technology [36,37]. The results indicated that ELP-ALS significantly enhanced solubility and improved enzymatic properties, including thermal and pH stability, storage stability, and substrate tolerance. Furthermore, the fused ELP-ALS enzyme was used to carry out the conversion of acetaldehyde into acetoin, and the final acetoin concentration of 1087.2 mM (95.75 g/L) was achieved by the fed-batch method. These results demonstrated the application potential of ELP-ALS as a promising biocatalyst for acetoin production from acetaldehyde due to its lower preparation cost, higher biocatalysis efficiency, and better stability.

## 2. Materials and Methods

### 2.1. Materials

The expression vector pET22b-*xyl*-ELPs, pET28a-*als* (I28V/L482E), and host strain *E. coli* BL21 (DE3) were stored at −80 °C in our lab. Chemical reagents including kanamycin, ampicillin, isopropyl-β-d-thiogalactoside (IPTG), acrylamide, TEMED, and Thiamine pyrophosphate (TPP) were purchased from Sigma Aldrich (Shanghai, China). SDS, Tris, 1-naphthol, ceatine, acetoin, and acetaldehyde were bought from Macklin Biotech (Shanghai, China). Glycerinum, glycine, acetic acid, methanol, ammonium persulfate, n-propyl alcohol, bromophenol blue, and Coomassie brilliant blue *R*250 were purchased from Sinopharma Chemical Reagent Co., Ltd. (Shanghai, China). Yeast extract powder and peptone were purchased from Thermo Fisher Scientific (Waltham, MA, USA). Bovine serum albumin was obtained from Solarbio Co. (Beijing, China). High-affinity nickel-nitrilotriacetic acid (Ni-NTA) resin was purchased from Genscript (Nanjing, China).

### 2.2. Construction and Expression of Fusion Protein

The linearized vector was obtained by double enzyme digestion with restriction enzymes *Nde* I and *Eco*R I to remove the *xyl* fragment encoding xylanase enzyme from vector pET22b-*xyl*-ELPs. Primers were designed according to the gene sequence of acetaldehyde lyase [29] and used to amplify the ALS:I28V/L482E fragment from pET28a-*als* (I28V/L482E). The recombinant expression plasmid pET22b-ELP-ALS (I28V/L482E) was obtained by homologous recombination to ligate the cloned fragment into linearized vector (Supplementary Materials, Tables S1 and S2). The resulting recombinant plasmid was then transformed into the *E. coli* BL21 (DE3) for the expression of fusion protein. For fusion protein expression, the recombinant *E. coli* harboring pET22b-ELP-ALS (I28V/L482E) was inoculated into Luria–Bertani (LB) media, containing 10 g/L of peptone, 5 g/L of yeast extract, and 10 g/L of NaCl with an addition of 100 μg/mL of ampicillin. The culture was incubated overnight at 37 °C and 180 rpm in an orbital shaker for seed culture preparation. Subsequently, 0.5 mL of seed culture was inoculated into 50 mL of fresh LB medium supplemented with 100 μg/mL ampicillin and was cultured at 37 °C and 180 rpm for 2–2.5 h. IPTG as an inducer was added to a final concentration of 0.5 mM, when the OD_600_ of cell density reached between 0.6 and 0.8, and the cells were induced at 18 °C for 24 h. The recombinant *E. coli* harboring pET28a-*als* (I28V/L482E) was cultured and induced using the same operation and conditions as the control. Induced cells were harvested by centrifuging at 8000 rpm and 4 °C for 20 min, then the cell pellets were resuspended in 10 mL phosphate buffer (50 mM, pH 8.0) and washed twice. Finally, the cells were resuspended in 10 mL precooled phosphate buffer and lysed by sonication (on ice) using an ultrasonic cell disruptor (JY 92-IIN, Scientz, Ningbo, China). The supernatants containing soluble ELP-ALS (I28V/L482E) or ALS (I28V/L482E) were separated from pellet by centrifugation at 12,000 rpm and 4 °C for 30 min. Portions of each sample (lysate, supernatant, and pellet) were saved for SDS-PAGE analysis. Protein concentration was determined by Bradford assay using bovine serum albumin as the standard.

### 2.3. Purification of Recombinant Protein

(1) Purification by Ni-NTA affinity chromatography (His-ALS (I28V/L482E))

The recombinant His-tagged protein His-ALS (I28V/L482E) was purified using Ni^2+^ affinity chromatography, as described in our previous study [38]. Briefly, recombinant *E. coli* cells expressing His-ALS (I28V/L482E) were resuspended in lysis buffer (50 mM sodium phosphate, 300 mM NaCl, 20 mM imidazole, pH 8.0) and lysed via sonication on ice. The lysate was centrifuged, and the supernatant containing the crude enzyme was incubated with Ni-NTA resin at 4 °C for 2 h to facilitate the binding of the His-tagged protein to the resin. Subsequently, the wash buffer (50 mM sodium phosphate, 300 mM NaCl, 50 mM imidazole, pH 8.0) flowed through the column to remove non-specific proteins. Finally, His-tagged protein His-ALS (I28V/L482E) was eluted from Ni-charged resin with elution buffer (50 mM sodium phosphate, 300 mM NaCl, 300 mM imidazole, pH 8.0) to obtain the pure His-ALS. The eluate containing the pure enzyme was desalted and concentrated using a 10 kDa cut-off Centriprep device (GE Health-care, Chicago, IL, USA). During the purification process, enzyme activity and protein concentration were assayed to analyze the enzyme recovery rate and purification fold.

(2) Purification by ITC (ELP-ALS (I28V/L482E))

The elastin-like polypeptide-fused ALS (ELP-ALS (I28V/L482E)) was purified using ITC technology. Recombinant *E. coli* cells were harvested by centrifugation at 4 °C and resuspended in PBS buffer (50 mM, pH 8.0). Cells were lysed by ultrasonic disruption on ice, and the supernatant containing crude enzymes was obtained by centrifugation. Sodium chloride was gradually added to the supernatant at room temperature until a final concentration of 3.0 M was achieved, followed by incubation for 20 min at room temperature. Then, the solution was centrifuged at 10,000 rpm for 15 min to collect precipitation. The supernatant was discarded and the precipitation was resuspended in pre-chilled PBS buffer on ice. Insoluble impurity was removed by centrifugation at 10,000 rpm for 10 min at 4 °C. The obtained supernatant was further purified using the repeated purification process to obtain pure enzymes. The enzyme recovery rate and purification fold were determined by measuring the changes in enzyme activity and protein concentration during the purification process to assess purification efficiency.

### 2.4. ALS Enzyme Activity Assay

The activities of His-ALS (I28V/L482E) and ELP-ALS (I28V/L482E) were assayed in a 0.5 mL reaction mixture containing 50 mM phosphate buffer (pH 8.0), 1 mM Mg^2+^, 0.1 mM TPP, and 100 mM acetaldehyde as substrate with 30 μg His-ALS (I28V/L482E) or 39 μg ELP-ALS (I28V/L482E) addition. The reaction was conducted at 30 °C for 1 h, and acetoin concentration from acetaldehyde was determined by the Voges–Proskauer (VP) reaction and calculated from the calibration curves of standard acetoin. A VP test containing diluted reaction supernatant (0.3 mL), 0.5% creatine (0.3 mL), 5% alpha-naphthol (0.3 mL), and 5% NaOH (0.3 mL) were sequentially mixed together and then reacted at 30 °C for 30 min. The OD_520_ of the reaction sample was measured by using a spectrophotometer. One unit of enzyme activity (U) was defined as the amount of enzyme required to produce 1 μmol acetoin in 1 min [32].

### 2.5. Determination of Optimal pH and Temperature

Optimum temperature and pH were determined using the same reaction mixture as described in enzyme activity assay section. The reaction mixtures with the purified His-ALS (I28V/L482E) and ELP-ALS (I28V/L482E) were incubated at various temperatures that ranged from 30 to 70 °C (pH 8.0) or in buffers over a pH range of 6.0–10.0 (30 °C) for 1 h. Then, the catalytic activity was assayed and compared as described above. The temperature and pH value resulting maximum enzyme activity were regarded as optimal temperature and pH, respectively.

### 2.6. Enzyme Stability and Substrate Tolerance

The enzyme characteristics including thermal stability, pH stability, storage stability, and substrate tolerance were investigated. To determine the thermal stability of the enzymes, 5 μM of the purified His-ALS and ELP-ALS were pre-incubated at temperatures of 20, 30, 40, 50, and 60 °C for 1 h, 4 h, and 12 h, respectively. Then, the residual enzyme activities were evaluated under its optimal temperature and pH value (30 °C and pH 7.0 for ELP-ALS, and 30 °C and pH 8.0 for His-ALS). The relative activity was calculated as a percentage of the initial activity assayed before pre-incubation, which represented 100% enzyme activity. To estimate pH stability, the enzyme was pre-incubated in buffers over a pH range of 6.0–10.0 at room temperature for 1 h, 4 h, and 12 h, respectively. Similarly, the residual enzyme activities and relative activity were calculated. For the storage stability evaluation of the purified enzymes, 20 μM of His-ALS and ELP-ALS (soluble and precipitated forms, the precipitated form was prepared with 3 M NaCl addition) were stored at defined temperatures (4 °C) for 24 days, and aliquots were taken every day for determining its residual activity. To determine the substrate tolerance of the enzymes, 100 μg His-ALS or 130 μg ELP-ALS (equal number of moles) were mixed with 0.5, 1.0, 1.5, 2.0 2.5, 3.0, 3.5, and 4.0 M acetaldehyde, respectively. Then, the system was incubated at 30 °C and 180 rpm for 6 h, and the acetoin yield and acetaldehyde conversion rate were calculated. The results presented are the averages of three independent experiments.

### 2.7. Kinetic Parameter Activity Assay

For the kinetic parameters assay, the enzymatic reactions of His-ALS and ELP-ALS were conducted using a gradient of acetaldehyde concentrations from 2 mM to 300 mM as substrate and were incubated under optimum temperature and pH conditions. One unit of ALS activity (U) was defined as the amount of enzyme required to produce 1 μmol acetoin in 1 min. The values of kinetic parameters (*K*_m_, *k*_cat_ and *k*_cat_/*K*_m_) were determined through the nonlinear regression fitting of the Michaelis–Menten equation. All assays were performed in triplicate.

### 2.8. Fed-Batch Strategy for Acetoin Production

The catalytic system was carried out in 5 mL reaction volume comprising 50 mM phosphate buffer (pH 7.0), 1 mM Mg^2+^, 0.1 mM TPP, and 15 μM purified enzyme, with substrate concentrations of 1 M or 2 M acetaldehyde under controlled conditions (30 °C and 180 rpm orbital agitation). Acetaldehyde concentrations in catalytic system were assayed every 1 h and were fed with a final concentration of 1.0 M or 2.0 M (same with the initial acetaldehyde concentration) into the reaction mixture when the acetaldehyde conversion rate exceeded 90%. The reaction mixtures were detected using a gas chromatograph system, as previously described [32].

## 3. Results

### 3.1. Expression of His-ALS and ELP-ALS

**Figure 1:**
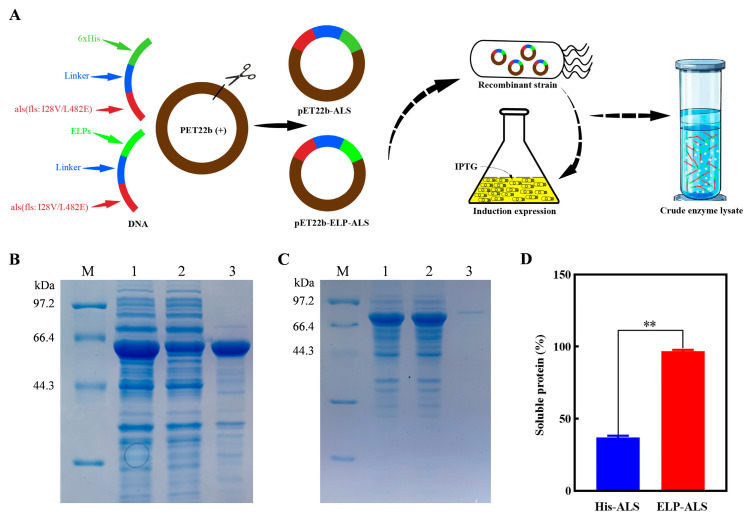
Heterologous expression, SDS-PAGE analysis, and comparative solubility profiling of recombinant His-ALS and fusion protein ELP-ALS. (**A**) Schematic representation of recombinant plasmid construction and heterologous expression in *E. coli* BL21 (DE3); (**B**) SDS-PAGE analysis of His-ALS expression and solubility (Lane M: protein marker; Lane 1: total cell lysate; Lane 2: soluble fraction; Lane 3: insoluble fraction); (**C**) SDS-PAGE analysis of ELP-ALS fusion protein expression and solubility (Lane M: protein marker; Lane 1: total cell lysate; Lane 2: soluble fraction; Lane 3: insoluble fraction); (**D**) Comparative solubility quantification of His-ALS and fusion protein ELP-ALS. ** Represented significant difference, *p* < 0.01.

The recombinant plasmids pET22b-ELP-ALS and pET22b-ALS were successfully constructed and transformed into the host strain *Escherichia coli* BL21 (DE3). Following IPTG induction, both His-ALS and the fusion protein ELP-ALS were efficiently expressed (Figure 1A). SDS-PAGE analysis confirmed distinct bands at molecular weights of 59.1 kDa (His-ALS) and 77.4 kDa (ELP-ALS), which were consistent with theoretical predictions based on their respective amino acid sequences. Solubility assessments demonstrated divergent behaviors: His-ALS predominantly accumulated as insoluble inclusion bodies, with only 36.9% soluble expression, whereas ELP-ALS exhibited markedly improved solubility (96.8% soluble expression) with minimal inclusion body formation (Figure 1B–D). These results indicated that fusion with ELPs significantly enhances the soluble expression of ALS in *E. coli*, thereby addressing a critical bottleneck in recombinant ALS enzyme production.

### 3.2. Purification of His-ALS and ELP-ALS

**Figure 2:**
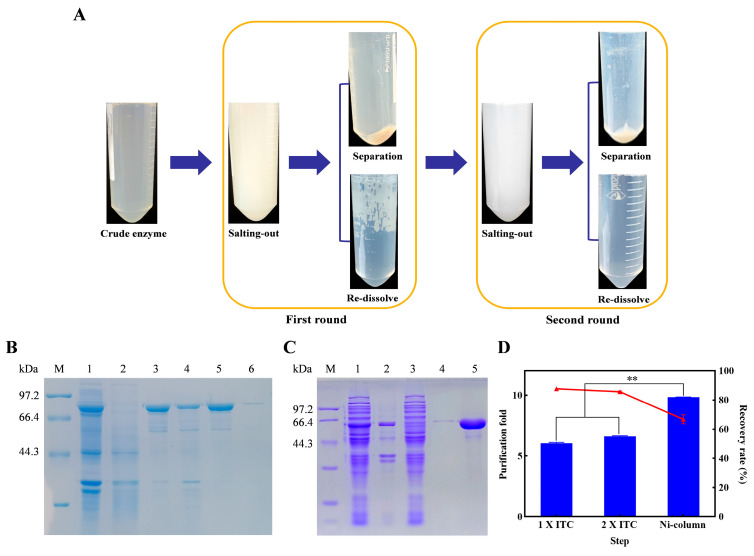
Purification strategy, SDS-PAGE analysis, and comparative efficiency of recombinant His-ALS and fusion protein ELP-ALS. (**A**) Schematic representation of fusion protein ELP-ALS purification via ITC; (**B**) SDS-PAGE analysis of ELP-ALS purification by ITC (Lane M: protein marker; Lane 1: crude enzyme lysate; Lane 2: supernatant from first salting-out; Lane 3: purified protein from first round of ITC; Lane 4: insoluble fraction from the first round of ITC; Lane 5: purified protein from second round of ITC; Lane 6: insoluble fraction from the second round of ITC); (**C**) SDS-PAGE analysis of His-ALS purification by Ni^2+^-affinity chromatography (Lane M: protein marker; Lane 1: crude enzyme lysate; Lane 2: insoluble fraction after cell ultrasonic disruption; Lane 3: flow-through fraction; Lane 4: wash buffer fraction; Lane 5: purified His-ALS); (**D**) Comparative purification efficiency metrics (purification fold and recovery rate) for His-ALS and ELP-ALS across separation and purification steps. ** Represented significant difference, *p* < 0.01.

The crude enzyme lysates containing His-ALS and fusion protein ELP-ALS were purified via Ni^2+^-affinity chromatography and ITC, respectively. The ITC workflow schematically was outlined in Figure 2A, which demonstrated ELP-ALS purification through two rounds of ITC. SDS-PAGE analysis (Figure 2B) confirmed the near-complete precipitation of ELP-ALS during the first ITC cycle, with no detectable target protein bands in the impurity-rich supernatant. The first round of ITC purification yielded ELP-ALS with high recovery rate (87.76%), and residual impurity necessitated a second round of ITC purification. Finally, the highly pure ELP-ALS was obtained with an enzyme recovery rate of 85.77%. The parallel purification of His-ALS via Ni-NTA (Figure 2C) generated a highly pure fraction, albeit with partial target protein lost in flowthrough and wash fractions. Comparative quantification (Figure 2D) revealed a 6.63-fold purity increase for ELP-ALS via ITC versus a 9.83-fold gain for His-ALS via Ni^2+^-affinity chromatography. The result was attributed to higher impurity proteins in the His-ALS lysate. Despite lower fold purification, ITC surpassed Ni^2+^-affinity chromatography in recovery rates (85.77% vs. 66.80%). Thus, it was used as a competitive method with superior yield and easy operation for recombinant protein purification.

### 3.3. Evaluation of the Effect of pH and Temperature

**Figure 3:**
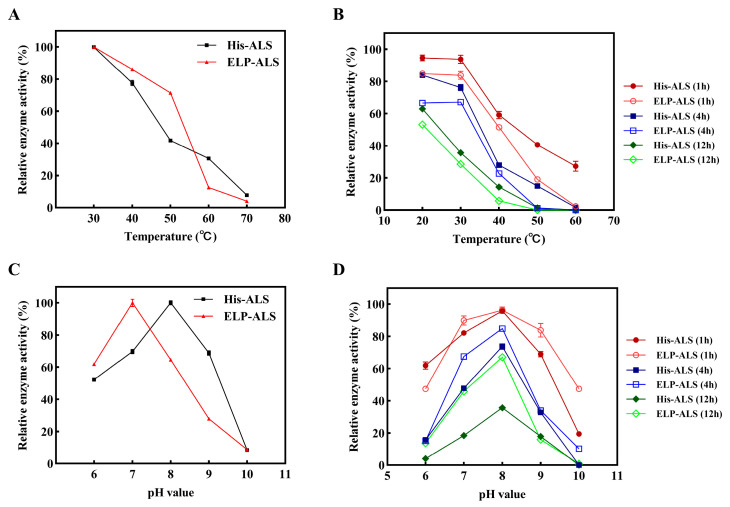
Comparative analysis of temperature and pH effects on the catalytic activity and stability of recombinant His-ALS and fusion protein ELP-ALS. (**A**) Optimal reaction temperature profiling for His-ALS and ELP-ALS; (**B**) thermal stability evaluation; (**C**) pH-dependent activity profiling for His-ALS and ELP-ALS; (**D**) pH stability evaluation.

The enzymatic activities of ELP-ALS and His-ALS were systematically evaluated under variable temperature and pH conditions. Both enzymes exhibited optimal catalytic activity at 30 °C, with activity declining progressively as temperatures increased. Notably, ELP-ALS was superior to His-ALS within the 30–50 °C range. However, its activity dropped sharply above 50 °C (Figure 3A). It was likely due to the temperature-induced aggregation of ELP’s amphiphilic chains beyond their transition temperature. Thermal stability assays revealed that both enzymes retained >80% residual activity after 1 h incubation below 30 °C, and enzyme activity decreased with the increasing of incubation temperatures. Comparative analysis indicated superior thermal stability for His-ALS relative to ELP-ALS (Figure 3B). pH profiling (6.0–10.0) at 30 °C demonstrated the optimal pH shift from 8.0 for His-ALS to 7.0 for ELP-ALS (Figure 3C). The assay of pH stability showed ELP fusion improved the pH stability of the ALS enzyme, which retained 67.05% activity after 12 h incubation at pH 8.0 compared to 35.65% for His-ALS (Figure 3D). These results collectively indicated that ELP fusion conferred superior enzymatic stability over histidine-tagged counterparts, particularly under suboptimal environmental conditions.

### 3.4. Storage Stability and Substrate Tolerance

**Figure 4:**
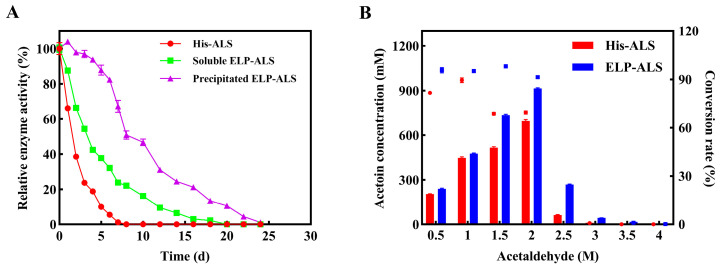
Storage stability (**A**) and substrate tolerance (**B**) assays of His-ALS and fusion protein ELP-ALS.

Comparative analysis revealed ELP fusion significantly enhanced ALS storage stability and substrate tolerance. During 4 °C storage (Figure 4A), His-ALS lost >50% activity within 1 day and was fully inactivated on the 7th day, while soluble ELP-ALS retained >50% activity until 3rd day (complete inactivation on the 20th day). Precipitated ELP-ALS showed superior stability and maintained 82.3% activity on the 6th day and nearly complete inactivation until the 24th day. Substrate tolerance assays demonstrated ELP-ALS’s enhanced adaptability, achieving conversion rate over 91% under 0.5~2.0 M acetaldehyde, while His-ALS dropped below 70% at 1.5 M and 2.0 M acetaldehyde. A rapid decrease in acetoin yield could be observed partially due to enzyme denaturation when the substrate concentration was over 2.5 M (Figure 4B). These results indicated that ELP fusion could efficiently improve the storage stability and substrate tolerance of the ALS enzyme.

### 3.5. Kinetic Parameters

The detailed kinetic analysis of purified His-ALS and ELP-ALS clearly demonstrated the catalytic advantages imparted by ELP fusion. The fusion protein ELP-ALS displayed a *V*_max_ of 15.25 U/mg, representing an 85.7% increase compared to His-ALS (8.21 U/mg). Comparative analysis using acetaldehyde as substrate, ELP-ALS exhibited a *k*_cat_ of 15.37 s^−1^, which was 1.82 times higher than that of His-ALS (8.45 s^−1^). Furthermore, ELP-ALS showed superior substrate affinity, as evidenced by its lower *K*_m_ value, leading to a catalytic efficiency *k*_cat_/*K*_m_ of 73.05 s^−1^·M^−1^—nearly threefold greater than the 24.63 s^−1^·M^−1^ observed for His-ALS (Table 1, Figure S1). These findings demonstrated that the strategic fusion of ELP improved the catalytic efficiency of ALS, thereby positioning ELP-ALS as a superior biocatalyst for scalable aldehyde lyase-dependent bioprocesses.

### 3.6. Fed-Batch Reaction

**Figure 5:**
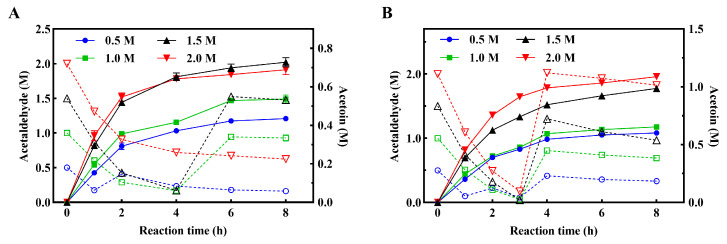
Fed-batch reaction of His-ALS (**A**) and fusion protein ELP-ALS (**B**) for acetoin production. The dashed and solid lines represented the changes of the substrate acetaldehyde and the product acetoin respectively during the biocatalytic process.

Herein, fed-batch reactions were performed to compare the catalytic performance of His-ALS and ELP-ALS across a range of acetaldehyde concentrations (0.5, 1.0, 1.5, 2 M). As shown in Figure 5A, His-ALS exhibited rapid initial substrate conversion, with supplementation timing varying by concentration: once at 1st h for 0.5 M systems, at 4th h for both 1.0 M and 1.5 M systems. The 2 M system required no supplementation and retained 0.62 M residual acetaldehyde substrate in total. The catalytic rate of His-ALS exhibited substantial concentration-dependent variations, where a moderate elevation in acetaldehyde concentration was shown to enhance acetoin biosynthesis. Optimal production was achieved at 1.5 M substrate concentration, yielding 0.73 M acetoin. In contrast, ELP-ALS demonstrated superior substrate utilization, necessitating earlier supplementation: twice (first hour and third hour) for 0.5 M systems and once (third hour) for higher concentrations. The catalytic performance of ELP-ALS showed pronounced concentration-dependent variation, with the 2.0 M acetaldehyde system achieving peak acetoin production (1.09 M). This output surpassed His-ALS by 49.32% as quantified in Figure 5B. These findings clearly demonstrate that ELP fusion enhances ALS’s catalytic efficiency and substrate tolerance. Thess advantages make it significantly more suitable for industrial biocatalytic applications in high-substrate-concentration environments.

## 4. Discussion

In our previous study, a multi-enzyme catalytic platform for bioethanol to acetoin conversion via cell-free catalysis was developed, wherein the suboptimal efficiency of FLS constituted a critical bottleneck. Rational engineering generated the FLS: I28V/L482E mutant (designated ALS herein), which demonstrated markedly improved catalytic capacity [32]. However, its limited solubility in *E. coli* expression systems and inefficient purification via Ni^2+^-affinity chromatography impeded practical implementation. To address these constraints, this study explored elastin-like polypeptide (ELP) fusion as a strategic modification. Heterologous expression vectors pET22b-ELP-ALS and pET22b-ALS were developed and expressed in *E. coli* (Figure 1A). Solubility analysis revealed that ELP fusion dramatically elevated soluble ALS expression from 36.9% to 96.8% (Figure 1B–D), which consisted with previous reports [36,39,40]. N-terminal residues play key roles in the stability, solubility, or function of the protein. N-terminal ELP fusion promotes proper folding by facilitating early domain release during translation process, thereby preventing the misfolding and suppressing aggregation of nascent polypeptides [41]. Moreover, the ELP moiety may exhibit chaperone-like functionality analogous to molecular chaperones such as tags MBP and TrxA, assisting correct protein folding and significantly enhancing solubility [42,43]. High-purity ELP-ALS was efficiently recovered through two rounds of ITC purification (Figure 2A). Although ITC exhibited lower purification fold than Ni^2+^-affinity chromatography due to more contaminant proteins in His-ELP crude lysates, it demonstrated the superior recovery efficiency of the target enzyme (Figure 2B–D) [44], establishing ELP-mediated ITC as a competitive alternative for enzyme purification.

ELP fusion proteins exhibit enhanced thermal stability and improved circulating half-life under harsh conditions, including thermal stress and denaturant exposure. This stability correlates with ELP chain length, as longer repeat sequences enable greater hydrogen bonding—a fundamental stabilizing mechanism in proteins [40]. In a study by Liu et al., fusion with collagen-like polypeptide (CLP) and ELP conferred significantly increased resistance to urea denaturation on SOD and DAAO [45]. Enzymatic characterization analysis revealed that ELP-ALS exhibited enhanced performance at moderate temperatures (30–50 °C), maintaining superior relative activity (Figure 3A). However, excessive temperatures induced ELP phase transition, causing abrupt activity loss. Thermal stability analysis showed reduced performance (Figure 3B), attributed to the temperature-dependent aggregation of amphiphilic ELP chains through hydrophilic block collapse during incubation, which compromised mass transfer efficiency [46]. The ELP fusion conferred significant pH adaptability, shifting the optimal pH of ELP-ALS from 8.0 towards acidity to pH 7.0 and significantly improved pH stability, particularly under acidic condition (Figure 3C,D). This establishes ELP as an effective acidic solubility tag that enhances enzymatic performance in acidic environments [47], consistent with previous reports on ELP’s stabilizing effects [48]. Application studies demonstrated remarkable storage stability, with precipitated ELP-ALS retaining 82.3% activity after 6 days at 4 °C (Figure 4A). This preservation is due to the protective encapsulation of active sites within protein aggregates, shielding them from environmental perturbations [49]. Notably, ELP fusion dramatically improved ALS substrate tolerance, enabling 91% conversion efficiency even at 2 M acetaldehyde concentration (Figure 4B). Collectively, these results demonstrate that the ELP tag enhances ALS structural robustness, improves thermal and pH stability, confers exceptional storage resilience in precipitated form, and maintains high catalytic efficiency under high-substrate concentrations, all of which represent critical advantages for industrial biocatalytic applications.

Enzyme kinetic analysis demonstrated that ELP-ALS exhibited significantly enhanced catalytic performance, displaying *V*_max_ and *k*_cat_/*K*_m_ values of 15.25 U/mg and 73.05 s^−1^·M^−1^, respectively. These values correspond to 1.86-fold and 2.97-fold improvements compared to His-ALS (Table 1), which was consistent with previous research by Peprah Addai et al. [44]. The results demonstrate that ELP fusion not only maintains ALS activity but substantially boosts its catalytic efficiency. Fed-batch reaction experiments further corroborated these enzymatic characteristics, showing that ELP-ALS maintains superior catalytic efficiency and demonstrates enhanced stability under high acetaldehyde concentrations. Finally, ELP-ALS produced 1.09 M acetoin in fed-batch reactions, representing a 49.32% yield improvement over His-ALS (Figure 5A,B). These findings collectively establish that ELP fusion synergistically enhances ALS’s catalytic efficiency, environmental resilience, and substrate tolerance while enabling chromatography-free purification through ELP’s reversible phase transition properties. These attributes establish ELP-ALS as an advanced biocatalyst for scalable aldehyde lyase-dependent bioprocesses.

## 5. Conclusions

In the present study, we reported that the fusion expression of ALS with ELPs dramatically enhanced the soluble expression, enzymatic stability, substrate tolerance, and catalytic efficiency of the ALS enzyme. Meanwhile, its purification process was shifted from chromatographic approaches to non-chromatographic ITC, which was suitable for scale-up enzyme preparation. A fed-batch reaction by ELP-ALS achieved 1.09 M acetoin output with a 49.32% yield improvement. These findings provided a promising biocatalyst for cost-effective and sustainable acetoin production from acetaldehyde.

## Figures and Tables

**Table 1:** Kinetic parameters of His-ALS and ELP-ALS.

Enzyme	*V*_max_ (U/mg)	*K*_m_ (mM) ^a^	*k*_cat_ (s^−1^) ^a^	*k*_cat_/*K*_m_ (s^−1^·M^−1^)
His-ALS: I28V/L482E	8.21 ± 1.72	343.84 ± 6.84	8.45 ± 0.26	24.63 ± 4.28
ELP-ALS: I28V/L482E	15.25 ± 3.10	210.39 ± 14.13	15.37 ± 3.12	73.05 ± 6.67

^a^ Data from three separate experiments are stated as the means ± SD (*n* = 3).

## Data Availability

The original contributions presented in this study are included in the article; further inquiries can be directed to the corresponding authors.

## Supplementary Materials

The following supporting information can be downloaded at: https://www.mdpi.com/article/10.3390/biom15091216/s1. Table S1. Plasmids and primers used in this study. Table S2. Gene sequence of ELPs and als(I28V/L482E); Figure S1. Enzyme kinetic curve of fusion protein ELP-ALS and His-ALS.

## Abbreviations

The following abbreviations are used in this manuscript:

ALS	Acetaldehyde lyase
ELPs	Elastin-like polypeptides
ITC	Inverse transition cycling
FLS	Formolase
Ni-NTA	Nickel-nitrilotriacetic acid
SDGs	Sustainable Development Goals
IPTG	Isopropyl-β-d-thiogalactoside
TEMED	Tetramethylethylenediamine
TPP	Thiamine pyrophosphate
SDS	Sodium dodecyl sulfate
Tris	Tris(hydroxymethyl)aminomethane
Xyl	Xylanase
LB	Luria–bertani
His	Histone
MBP	Maltose-Binding Protein
TrxA	Thioredoxin A
CLP	Collagen-like polypeptide
SOD	Superoxide dismutase
DAAO	D-amino acid oxidase

## References

[1] Jiang Y., Woortman A.J., van Ekenstein G.O., Loos K. (2013). Enzyme-catalyzed synthesis of unsaturated aliphatic polyesters based on green monomers from renewable resources. Biomolecules.

[2] Becker M., Nikel P., Andexer J.N., Lütz S., Rosenthal K. (2021). A Multi-Enzyme Cascade Reaction for the Production of 2′3′-cGAMP. Biomolecules.

[3] Alcántara A.R., Domínguez de María P., Littlechild J.A., Schürmann M., Sheldon R.A., Wohlgemuth R. (2022). Biocatalysis as Key to Sustainable Industrial Chemistry. ChemSusChem.

[4] Amatto I.V.D.S., Rosa-Garzon N.G.D., Simões F.A.D.O., Santiago F., Leite N.P.D.S., Martins J.R., Cabral H. (2022). Enzyme engineering and its industrial applications. Biotechnol. Appl. Biochem..

[5] Wu S., Snajdrova R., Moore J.C., Baldenius K., Bornscheuer U.T. (2021). Biocatalysis: Enzymatic Synthesis for Industrial Applications. Angew. Chem. Int. Ed. Engl..

[6] Maina S., Prabhu A.A., Vivek N., Vlysidis A., Koutinas A., Kumar V. (2022). Prospects on bio-based 2,3-butanediol and acetoin production: Recent progress and advances. Biotechnol. Adv..

[7] Xiao Z., Lu J.R. (2014). Generation of acetoin and its derivatives in foods. J. Agric. Food. Chem..

[8] Zhou C., Shen H., Yan S., Ma C., Leng J., Song Y., Gao N. (2024). Acetoin Promotes Plant Growth and Alleviates Saline Stress by Activating Metabolic Pathways in Lettuce Seedlings. Plants.

[9] Qin Y., Han Y., Shang Q., Li P. (2015). Complete genome sequence of *Bacillus amyloliquefaciens* L-H15, a plant growth promoting rhizobacteria isolated from cucumber seedling substrate. J. Biotechnol..

[10] Li T., Liu P., Guo G., Liu Z., Zhong L., Guo L., Chen C., Hao N., Ouyang P. (2023). Production of acetoin and its derivative tetramethylpyrazine from okara hydrolysate with *Bacillus subtilis*. AMB Express.

[11] Cui Z., Wang Z., Zheng M., Chen T. (2022). Advances in biological production of acetoin: A comprehensive overview. Crit. Rev. Biotechnol..

[12] Zhu P., Zhang C., Chen J., Zeng X. (2024). Multilevel systemic engineering of *Bacillus licheniformis* for efficient production of acetoin from lignocellulosic hydrolysates. Int. J. Biol. Macromol..

[13] Xiao Z., Lu J.R. (2014). Strategies for enhancing fermentative production of acetoin: A review. Biotechnol. Adv..

[14] Cui Z., Zheng M., Ding M., Dai W., Wang Z., Chen T. (2023). Efficient production of acetoin from lactate by engineered *Escherichia coli* whole-cell biocatalyst. Appl. Microbiol. Biotechnol..

[15] Bae S.J., Kim S., Park H.J., Kim J., Jin H., Kim B.G., Hahn J.S. (2021). High-yield production of (R)-acetoin in *Saccharomyces cerevisiae* by deleting genes for NAD(P)H-dependent ketone reductases producing meso-2,3-butanediol and 2,3-dimethylglycerate. Metab. Eng..

[16] Li S., Liu L., Chen J. (2015). Compartmentalizing metabolic pathway in *Candida glabrata* for acetoin production. Metab. Eng..

[17] Moxley W.C., Brown R.E., Eiteman M.A. (2023). *Escherichia coli aceE* variants coding pyruvate dehydrogenase improve the generation of pyruvate-derived acetoin. Eng. Life. Sci..

[18] Zhang L., Xu Q., Zhan S., Li Y., Lin H., Sun S., Sha L., Hu K., Guan X., Shen Y. (2014). A new NAD(H)-dependent meso-2,3-butanediol dehydrogenase from an industrially potential strain *Serratia marcescens* H30. Appl. Microbiol. Biotechnol..

[19] Rehman S., Islam M.K., Khanzada N.K., Kyoungjin An A., Chaiprapat S., Leu S.Y. (2021). Whole sugar 2,3-butanediol fermentation for oil palm empty fruit bunches biorefinery by a newly isolated *Klebsiella pneumoniae* PM2. Bioresour. Technol..

[20] Lu L., Mao Y., Kou M., Cui Z., Jin B., Chang Z., Wang Z., Ma H., Chen T. (2020). Engineering central pathways for industrial-level (3*R*)-acetoin biosynthesis in *Corynebacterium glutamicum*. Microb. Cell. Fact..

[21] Maina S., Schneider R., Alexandri M., Papapostolou H., Nychas G.J., Koutinas A., Venus J. (2021). Volumetric oxygen transfer coefficient as fermentation control parameter to manipulate the production of either acetoin or D-2,3-butanediol using bakery waste. Bioresour. Technol..

[22] Tsigoriyna L., Petrova P., Petrov K. (2023). High production of acetoin from glycerol by *Bacillus subtilis* 35. Appl. Microbiol. Biotechnol..

[23] Zhang J., Zhao J., Fu Q., Liu H., Li M., Wang Z., Gu W., Zhu X., Lin R., Dai L. (2024). Metabolic engineering of *Paenibacillus polymyxa* for effective production of 2,3-butanediol from poplar hydrolysate. Bioresour. Technol..

[24] Meng D., Wei X., Bai X., Zhou W., You C. (2020). Artificial in Vitro Synthetic Enzymatic Biosystem for the One-Pot Sustainable Biomanufacturing of Glucosamine from Starch and Inorganic Ammonia. ACS Catal..

[25] Zhao Q., Ansorge-Schumacher M.B., Haag R., Wu C. (2020). Living whole-cell catalysis in compartmentalized emulsion. Bioresour. Technol..

[26] Muñoz-Sánchez D., Carceller A., Álvaro G., Romero O., Guillén M. (2025). Artificial cell-free system for the sustainable production of acetoin from bioethanol. Bioresour. Technol..

[27] Jia X., Kelly R.M., Han Y. (2018). Simultaneous biosynthesis of (*R*)-acetoin and ethylene glycol from D-xylose through in vitro metabolic engineering. Metab. Eng. Commun..

[28] Jia X., Liu Y., Han Y. (2017). A thermophilic cell-free cascade enzymatic reaction for acetoin synthesis from pyruvate. Sci. Rep..

[29] Siegel J.B., Smith A.L., Poust S., Wargacki A.J., Bar-Even A., Louw C., Shen B.W., Eiben C.B., Tran H.M., Noor E. (2015). Computational protein design enables a novel one-carbon assimilation pathway. Proc. Natl. Acad. Sci. USA.

[30] Peng K., Guo D., Lou Q., Lu X., Cheng J., Qiao J., Lu L., Cai T., Liu Y., Jiang H. (2020). Synthesis of Ligustrazine from Acetaldehyde by a Combined Biological-Chemical Approach. ACS. Synth. Biol..

[31] Zhang L., Singh R., Sivakumar D., Zewang G., Li J., Chen F., He Y., Guan X., Kang Y., Lee J.-K. (2017). An artificial synthetic pathway for acetoin, 2,3-butanediol, and 2-butanol production from ethanol using cell free multi-enzyme catalysis. Green Chem..

[32] Zhang J., Lin H., Zheng C., Yang B., Liang M., Lin Y., Zhang L. (2024). Efficient 2,3-Butanediol Production from Ethanol by a Modified Four-Enzyme Synthetic Biosystem. Molecules.

[33] Tian X., Feng M., Wei X., Cheng C., He K., Jiang T., He B., Gu Z. (2024). In situ formed depot of elastin-like polypeptide-hirudin fusion protein for long-acting antithrombotic therapy. Proc. Natl. Acad. Sci. USA.

[34] Yeboah A., Cohen R.I., Rabolli C., Yarmush M.L., Berthiaume F. (2016). Elastin-like polypeptides: A strategic fusion partner for biologics. Biotechnol. Bioeng..

[35] Mills C.E., Michaud Z., Olsen B.D. (2018). Elastin-like Polypeptide (ELP) Charge Influences Self-Assembly of ELP-mCherry Fusion Proteins. Biomacromolecules.

[36] Du K., Sun J., Song X., Song C., Feng W. (2015). Enhancement of the solubility and stability of D-amino acid oxidase by fusion to an elastin like polypeptide. J. Biotechnol..

[37] Han J., Fang S., He X., Wang L., Li C., Wu J., Cai Y., Wang Y. (2022). Combination of aqueous two-phase flotation and inverse transition cycling: Strategies for separation and purification of recombinant β-glucosidase from cell lysis solution. Food. Chem..

[38] Lin Y., Li C., Wei C., Lin H., Zhang L. (2024). Mining, Identification, and Characterization of Three Xylanases from the Microbiota of *T. fuciformis* with Its Companion Strains. Catalysts.

[39] Trabbic-Carlson K., Liu L., Kim B., Chilkoti A. (2004). Expression and purification of recombinant proteins from *Escherichia coli*: Comparison of an elastin-like polypeptide fusion with an oligohistidine fusion. Protein Sci..

[40] Zhou Y., Li X., Yan D., Addai Peprah F., Ji X., Fletcher E.E., Wang Y., Wang Y., Gu J., Lin F. (2019). Multifunctional elastin-like polypeptide renders β-glucosidase enzyme phase transition and high stability. Biotechnol. Biofuels.

[41] Dall N.R., Mendonça C., Torres Vera H.L., Marqusee S. (2024). The importance of the location of the N-terminus in successful protein folding in vivo and in vitro. Proc. Natl. Acad. Sci. USA.

[42] Tang N.C., Su J.C., Shmidov Y., Kelly G., Deshpande S., Sirohi P., Peterson N., Chilkoti A. (2024). Synthetic intrinsically disordered protein fusion tags that enhance protein solubility. Nat. Commun..

[43] Zou Z., Cao L., Zhou P., Su Y., Sun Y., Li W. (2008). Hyper-acidic protein fusion partners improve solubility and assist correct folding of recombinant proteins expressed in *Escherichia coli*. J. Biotechnol..

[44] Peprah Addai F., Wang T., Kosiba A.A., Lin F., Zhen R., Chen D., Gu J., Shi H., Zhou Y. (2020). Integration of elastin-like polypeptide fusion system into the expression and purification of *Lactobacillus* sp. B164 β-galactosidase for lactose hydrolysis. Bioresour. Technol..

[45] Liu D., Du K., Feng W. (2018). Immobilization of enzymes using a multifunctional fusion polypeptide. Biotechnol. Lett..

[46] Qin G., Perez P.M., Mills C.E., Olsen B.D. (2016). Effect of ELP Sequence and Fusion Protein Design on Concentrated Solution Self-Assembly. Biomacromolecules.

[47] Paraskevopoulou V., Falcone F.H. (2018). Polyionic Tags as Enhancers of Protein Solubility in Recombinant Protein Expression. Microorganisms.

[48] Fu Y., Mao S., Liao T., Feng W. (2025). Simultaneous production of linear α-olefins and 2,5-furandicarboxylic acid by combining two recombinant enzymes OleT-ELP and HMFO-ELP. Enzyme Microb. Technol..

[49] Chen N., Chang B., Shi N., Yan W., Lu F., Liu F. (2023). Cross-linked enzyme aggregates immobilization: Preparation, characterization, and applications. Crit. Rev. Biotechnol..

