# Supplementary material for: Improvement of Soluble Expression, Stability, and Activity of Acetaldehyde Lyase by Elastin-like Polypeptides Fusion for Acetoin Production from Acetaldehyde
Source: Biomolecules. 2025 Aug 22;15(9):1216. doi: 10.3390/biom15091216 (PMC12467592; doi:10.3390/biom15091216)
Supplement: Supplementary file 1 [file biomolecules-15-01216-s001.zip › biomolecules-3791851-supplementary.pdf]

biomolecules

MDPI

Table S1 Plasmids and primers used in this study

Plasmids and primers	Describe	Note
Primer Als-forward	TAAGAAGGAGATATACATATGGC GATGATTACAGGCGGCGAAC	For *als*(I28V/L482E) amplification
Primer Als-reverse	GAGCCGCTGGATCCGAATTCTGC GAAGGGGTCCATGCCGATC	For *als*(I28V/L482E) amplification
Plasmid pET22b-*xyl*-ELPs	pET28a origin, T7 promoter, *xyl*, *ELPs*, amp^R	linearized vector pET22b-ELPs by double digestion
Plasmid pET28a-*als*(I28V/L482E)	pET28a origin, T7 promoter, *als*(I28V/L482E), kan^R	Template of *als*(I28V/L482E) gene
Plasmid pET22b-ELP-ALS (I28V/L482E)	pET28a origin, T7 promoter, *ELPs*, *als*(I28V/L482E),amp^R	Plasmid construction in this study

Table S2 Gene sequence of ELPs and *als*(I28V/L482E)

ELPs	GTACCGGGTAAAGGTGTTCCTGGCGTGGGTGTTCCGGGCGTAGGTGTCCCAGGTGTGGGCG-<br>TACCGGGCGTTGGTGTTCCTGGTGTCGGCGTACCGGGCGTGGGTGTACCAGGTGTGGGCGTTCCGGG<br>TTTCGGCGTGCCGGGCGTGGGCGTACCGGGTAAAGGTGTTCCTGGCGTGGGTGTTCCGGGCG-<br>TAGGTGTCCCAGGTGTGGGCGTACCGGGCGTTGGTGTTCCTGGTGTCGGCGTGCCGGGCGTGGGTGT<br>ACCAGGTGTGGGCGTTCCGGGTTTCGGCGTGCCGGGCGTGGGCGTACCGGG-<br>TAAAGGTGTTCCTGGCGTGGGTGTTCCGGGCGTAGGTGTCCCAGGTGTGGGCGTACCGGGCGTTGGT<br>GTTCCTGGTGTCGGCGTACCGGGCGTGGGTGTAC-<br>CAGGTGTGGGCGTTCCGGGTTTCGGCGTGCCGGGCGTGGGCGTACCGGGTAAAGGTGTTCCTGGCGT<br>GGGTGTTCCGGGCGTAGGTGTCCCAGGTGTGGGCGTACCGGGCGTT-<br>GGTGTTCCTGGTGTCGGCGTGCCGGGCGTGGGTGTACCAGGTGTGGGCGTTCCGGGTTTCGGCGTGC<br>CGGGCTGGCCG
als(I28V/L482E)	ATGGCGATGATTACAGGCGGCGAACTGGTTGTTCGCACCCTAA-<br>TAAAGGCTGGGGTCGAACATCTGTTCGGCCTGCACGGCGTGCATATCGATACGATTTTTCAAGCCTG<br>TCTCGATCATGATGTGCCGATCATCGACACCCGCCATGAGGCCGCCGCAGGG-<br>CATGCGGCCGAGGGCTATGCCCGCGCTGGCGCCAAGCTGGGCGTGGCGCTGGTCACGGCGGGCGGG<br>GGATTTACCAATGCGGTCACGCCCATTGCCAACGCTCGTACCGATCGCAC-<br>GCCGGTGCTCTTCCTCACCGGATCGGGCGCGCTGCGTGATGATGAAACCAACACGTTGCAGGCGGG<br>GATTGATCAGGTCGCCATGGCGGCGCCCATTACCAAATGGGCGCATCGGGTGATGGCAAC-<br>CGAGCATATCCCACGGCTGGTGATGCAGGCGATCCGCGCCGCGTTGAGCGCGCCACGCGGGCCGGT<br>GTTGCTGGATCTGCCGTGGGATATTCTGATGAACCAGATTGATGAGGA-<br>TAGCGTCATTATCCCCGATCTGGTCTTGTCCGCGCATGGGGCCCATCCCGACCCTGCCGATCTGGATC<br>AGGCTCTCGCGCTTTTGCGCAAGGCGGAGCGGCCGGTCATCGTGCTCGGCTCAGAA-<br>GCCTCGCGGACAGCGCGCAAGACGGCGCTTAGCGCCTTCGTGGCGGCGACTGGCGTGCCGGTGTTT<br>GCCGATTATGAAGGGCTAAGCATGCTCTCGGGGCTGCCCGATGC-<br>TATGCGGGGCGGGCTGGTGCAAAACCTCTATTCTTTTGCCAAAGCCGATGCCGCGCCAGATCTCGTG<br>CTGATGCTGGGGGCGCGCTTTGGCCTTAACACCGGGCATGGATCTGGGCAGTTGATCCCCCA-<br>TAGCGCGCAGGTCATTCAGGTCGACCCTGATGCCTGCGAGCTGGGACGCCTGCAGGGCATCGCTCTG<br>GGCATTGTGGCCGATGTGGGTGGGACCATCGAGGCTTTGGCGCAGGCCACCGCGCAA-<br>GATGCGGCTTGGCCGGATCGCGGCGACTGGTGCGCCAAAGTGACGGATCTGGCGCAAGAGCGCTAT<br>GCCAGCATCGCTGCGAAATCGAGCAGCGAGCATGCGCTCCACCCCTTTCACGCCTCG-<br>CAGGTCATTGCCAAACACGTCGATGCAGGGGTGACGGTGGTAGCGGATGGTGGCCTGACCTATCTCT<br>GGCTGTCCGAAGTGATGAGCCGCGTGAAACCCGGCGGTTTTCTCTGCCACGGC-<br>TATCTAAACTCGATGGGCGTGGGCTTCGGCACGGCGCTGGGCGCGCAAGTGGCCGATCTTGAAGCA<br>GGCCGCCGCACGATCCTTGTGACCGGCGATGGCTCGGTGGGCTATAGCATCGGTGAATTTGA-<br>TACGCTGGTGCGCAAACAATTGCCGCTGATCGTCATCATCATGAACAACCAAAGCTGGGGGTGGAC<br>AGAACATTTCCAGCAATTGGCCGTCGGCCCCAATCGCGTGACGGGCACCCGTTT-<br>GGAAAATGGCTCCTATCACGGGGTGGCCGCCGCCTTTGGCGCGGATGGCTATCATGTCGACAGTGTG<br>GAGAGCTTTTCTGCGGCTCTGGCCCAAGCGCTCGCCCATAATCGCCCCGCCTG-<br>CATCAATGTCGCGGTCGCGCTCGATCCGATCCCGCCCGAAGAACTCATTCTGATCGGCATGGACCCC<br>TTCGCATGA

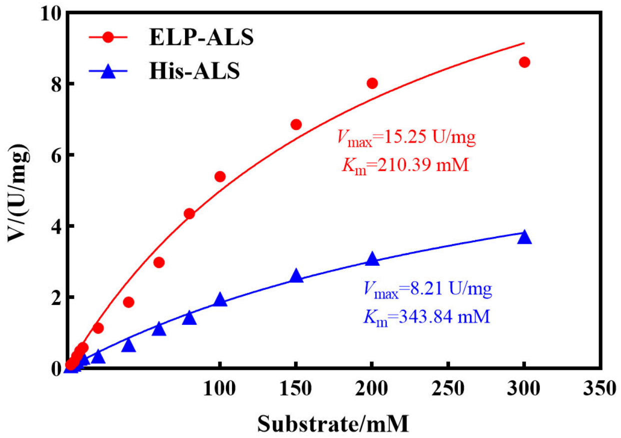

Figure S1. Enzyme kinetic curve of fusion protein ELP-ALS and His-ALS
